# Mammographic images segmentation based on chaotic map clustering algorithm

**DOI:** 10.1186/1471-2342-14-12

**Published:** 2014-03-25

**Authors:** Marius Iacomi, Donato Cascio, Francesco Fauci, Giuseppe Raso

**Affiliations:** 1 Dipartimento di Fisica e Chimica, Università Degli Studi di Palermo, Palermo, Italy; 2 Institutul de Ştiinţe Spaţiale, Bucharest, Măgurele, Romania

**Keywords:** Chaotic maps, Clustering algorithms, Cooperative behavior, Segmentation, Mammography, Features, Mass lesions, Microcalcifications, Breast cancer

## Abstract

**Background:**

This work investigates the applicability of a novel clustering approach to the segmentation of mammographic digital images. The chaotic map clustering algorithm is used to group together similar subsets of image pixels resulting in a medically meaningful partition of the mammography.

**Methods:**

The image is divided into pixels subsets characterized by a set of conveniently chosen features and each of the corresponding points in the feature space is associated to a map. A mutual coupling strength between the maps depending on the associated distance between feature space points is subsequently introduced. On the system of maps, the simulated evolution through chaotic dynamics leads to its natural partitioning, which corresponds to a particular segmentation scheme of the initial mammographic image.

**Results:**

The system provides a high recognition rate for small mass lesions (about 94% correctly segmented inside the breast) and the reproduction of the shape of regions with denser micro-calcifications in about 2/3 of the cases, while being less effective on identification of larger mass lesions.

**Conclusions:**

We can summarize our analysis by asserting that due to the particularities of the mammographic images, the chaotic map clustering algorithm should not be used as the sole method of segmentation. It is rather the joint use of this method along with other segmentation techniques that could be successfully used for increasing the segmentation performance and for providing extra information for the subsequent analysis stages such as the classification of the segmented ROI.

## Background

At present, breast cancer is the most common cancer among women, after cancers of the skin, and the second leading cause of cancer death in women after lung cancer [1-3]. The most widely used method for detecting breast cancer in its early stages is the mammography, a technique which has lately taken advantage of the supplementary features offered by the digital format [1]. During the last decades, the automatic detection of pathologies in the mammographic images has became a widespread auxiliary technique in radiology and the CAD (Computer Aided Detection) systems have proven their effectiveness mostly as a “second reader” (see [4-6]). The partitioning of the image in medically meaningful components (homogeneous with respect to one or several appropriately chosen characteristics) is a compulsory step in the process of automatic searching of pathologies in the images [7-11]. This phase plays a crucial role [12]: any non segmented lesion at this stage will be irremediably lost for any further analysis. While a wide variety of segmentation approaches have been proposed, there is no standard algorithm that can ensure high levels of accuracy for all imaging applications [13-15]. Furthermore, many segmentation methods rely on specific testing on an actual database [16] and the performance depends on database specificities. In particular, the segmentation of mass lesions in mammographies remains a challenging task since the masses are usually embedded and obscured by surrounding normal breast parenchyma [1,17]. The segmentation methods proposed in mammography and more generally in medical imaging span a broad range of techniques, see e.g. [18] for a recent review and [19,20] for particular examples. One of the generic segmentation approaches proposed more than three decades ago is the feature-based clustering method [21], which associates to each pixel or group of pixels from the image a set of appropriately chosen numerical parameters and transforms the primary segmentation task in a derived clustering problem in the associated feature space. Within this approach, the process of feature clustering becomes the crucial part of the segmentation algorithm. The main advantage of this approach is that the method does not require the use of a training set [15]. Towards the end of the last century, a new promising nonparametric method of clustering relying on the physical properties of the inhomogeneous Potts model has been proposed by Blatt, Wiseman and Domany [22]; a similar approach was proposed in terms of coupled chaotic dynamical networks by Manrubia and Mikhalkov [23] and has been further refined and restated with coupled chaotic maps by L. Angelini et al. [24]. During the last decade, a series of successful applications of this clustering method has emerged in the literature, such as landmine detection [25], EEG signals analysis in medicine [26] or financial analysis (stock markets [27], financial time series [28]). On the other hand, the chaotic map based algorithms have been proposed in many other contexts such as analysis of matrix metalloproteinases [29] or the medical image encryption technology [30]. The wide applicability of the feature clustering with coupled chaotic maps inspired us to investigate its effectiveness in the case of mammographic images with their specific characteristics. This paper focuses on the application of the chaotic maps clustering method for the segmentation of digital mammographic medical images. The results of this application are subsequently presented.

## Methods

**Figure 1:**
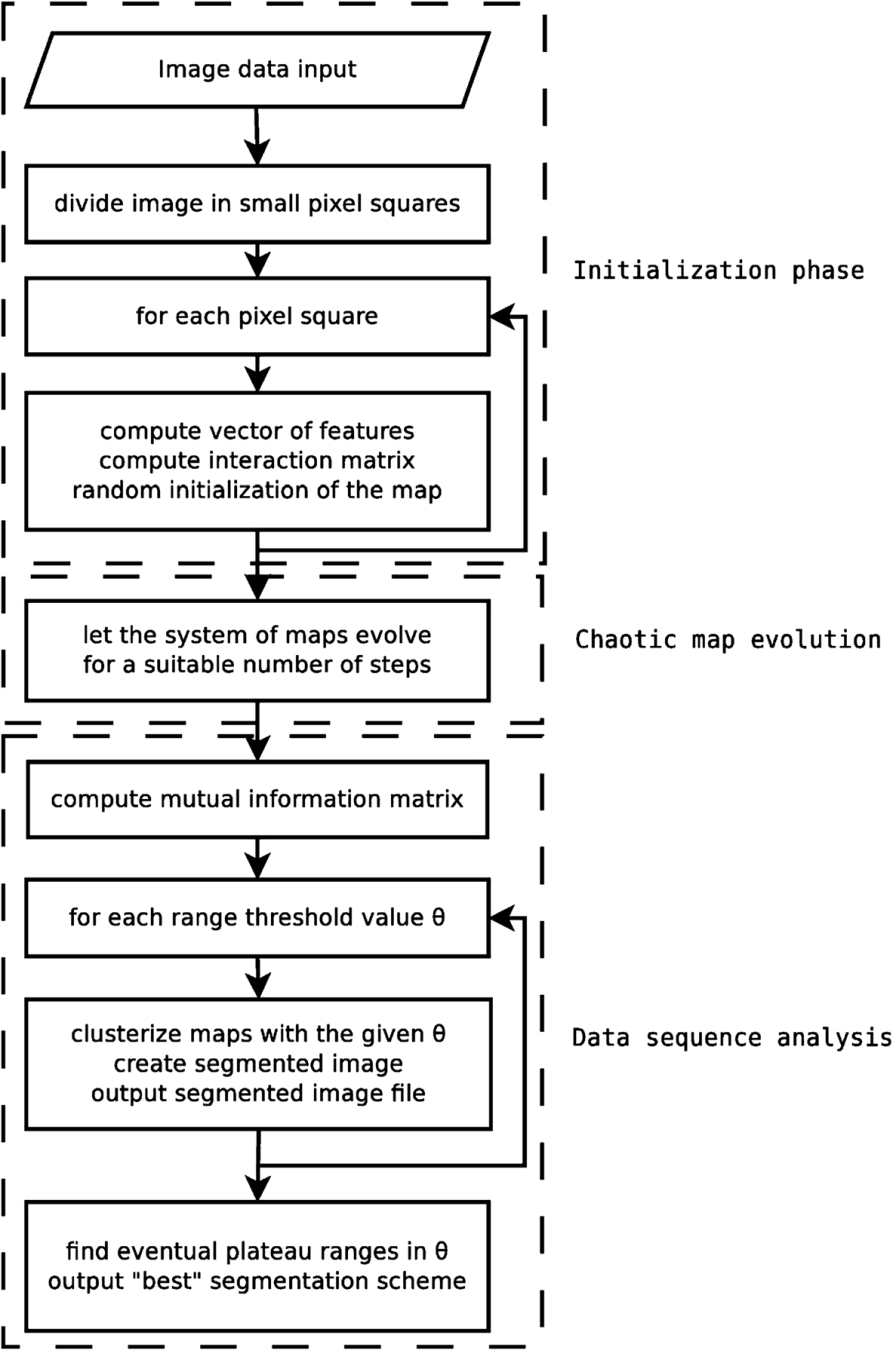
**Flowchart of the chaotic map segmentation algorithm.** The flowchart of the chaotic map segmentation algoritm used within this work. Its three phases are delimited by dashed lines.

The chaotic map clustering method has been thoroughly described in several references ([24-27]); the reader is therefore invited to consult them for more details concerning the method. For completeness, we present here a sketch of the method we have used, following the general flowchart in reference [26]. The proposed method consists of three major phases (see the flowchart in Figure 1).

In the initialization phase, the mammographic image to be analyzed is divided into elementary units of pixels (squares) and a feature vector is computed for each elementary unit. A dynamical variable is also associated with each unit and initialized at random. Finally, an “interaction coefficient” is computed for each pair of units.

The second phase is the core of the method as it features the basic idea of chaotic map clustering: the integration of a dynamical system in the feature space. For each point in the feature space, the associated dynamical variable is allowed to iteratively evolve according to a functional law corresponding to the distance matrix. In mathematical terms, for each point in the feature space {***r***_***i***_}, one defines a real dynamical variable *x*_i_ ∈ [−1,1] (*i* labels the data points). For two points *i* and *j*, the “interaction” matrix element is defined as

$$J_{i\;j}=\exp\;\left[-{\left(r_{i}–r_{j}\right)}^{2}/2a^{2}\right]$$

where *a* is a local scale parameter. The iterative evolution law is given by

$$x_{i}\left(t+1\right)=\left(\sum_{j\neq i}\;J_{i\;j}\;f\left(x_{j}\left(t\right)\right)\right)/C_{i}$$

where

$$C_{i}=\sum_{j\neq i}\;J_{i\;j}$$

and

$$f\left(x\right)=1-2x^{2}$$

is the usual logistic map which is at the origin of the chaotic dynamics of the system. The local length scale *a* is estimated as average distance of the *K*-nearest neighbors (KNN), where *K* is the only free parameter of the algorithm.

The third phase is the analysis of the time evolution of the coupled chaotic map system. The trajectories of the associated maps exhibit a more or less synchronized behavior depending on how close are the corresponding points in the feature space irrespective of the randomly chosen initial state of the maps: the closer are the representative points, the more similar are the trajectories. Since the maps are chaotic, there is no final stationary regime. Hence, to evaluate mutual correlations one has to operate a cut-off after a large enough number of iterations [24]. In order to define a meaningful measure for the actual synchronism of pairs of maps, one extracts the time sequence *S*_*i*_ (*t*) of the sign bits corresponding to the map *x*_*i*_ (*t*) as *S*_*i*_ (*t*) = 1 if *x*_*i*_ (*t*) > 0 and *S*_*i*_ (*t*) = 0 otherwise, and one computes on this basis the value of the mutual information as:

$$I_{i\;j}=H_{i}+H_{j}–H_{i\;j}$$

where *H*_*i*_ is the Boltzmann entropy for the *i*-th map sequence and *H*_*i j*_ is the joint entropy of the maps *i* and *j*. The mutual information provides a good measure of the synchronism [31], and it ranges between 0 for completely non-correlated maps and *ln* 2 for exactly synchronized maps. All the pairs of maps for which the mutual information exceeds a threshold θ are considered connected and the corresponding points in the feature space are assumed to belong to a same cluster. Thus, each value of the threshold θ defines a clustering of the data points. The number of clusters monotonically increases with the threshold and their hierarchy is naturally obtained from the graph’s increasing connectivity. For θ = 0 all data points belong to a single cluster while for θ = *ln* 2 the partitioning will consist in one cluster for each point. Between these extreme values lays the “best” partitioning scheme whose optimality is identified by its maximal stability when varying θ. The stability conditions can be imposed on the number of clusters and on the size of the biggest clusters. These conditions are strong indications that the clustering scheme obtained through application of the algorithm is not a spurious artifact of meaningless numerical output but it rather reflects some deeper similarity property of the input data.

The method has been implemented in order to take as input the data points corresponding to the digital mammographic images to be segmented. The computation begins with the partition of the image into squares small enough to match the typical dimensionality of the smallest objects of interest for the radiologist and rich enough in pixels in order to enable the computation of relevant associated features. In our experiments the side of the square usually ranged around 20 pixels. For each square a vector of features is computed leading to an associated data point in the feature space. Due to the fixed geometry of the initial partitioning, no geometrical or form-based feature can be taken into account at this stage. The position of the square in the image has a definite importance: any segmented lesion should be a contiguous region composed of one or several groups of pixels, therefore any medically meaningful clusters of points in the feature space have to correspond to spatially connected groups of neighbor squares in the image. Hence, it results necessary to treat separately the positional feature (the *x* and *y* of a data point) as a compulsory check. Other features used are the usual statistical central moments (mean pixel gray value, variance, kurtosis, skewness) and several autocorrelation values (such as energy, entropy, contrast) accounting for the texture (see [32-34] for other generic texture features and [35-38] for mammographic specific features).

The values of features have been linearly normalized to zero mean and unit variance over the whole image set of points as in [39]. Furthermore, a Karhunen-Loève transformation has been subsequently used in order to eliminate redundancies and focus the analysis on the main independent components of the feature vectors.

In order to make a more meaningful evaluation of the eventual gains of applying the chaotic maps method, the clusters have been also obtained in an alternative manner, by using the simple Euclidean distance in the feature space between the pairs of squares rather than the mutual information.

The clusters obtained on this basis are visualized as different gray-level regions on the image. Due to the border effects, the contour of the breast usually introduces a series of spurious clusters with no real meaning. In our analysis, we have chosen to cut-off these artifacts by default assigning a strip of border pixel squares to the unique border cluster; the choice has the advantage that the breast shape contour is immediately visible on the segmented image (in white), while exhibiting low probability to cut-off also eventual pathologies, usually found more in depth.

The mammographic image database used for this study consists of a group of 149 selected cases for a total of 298 images. More specifically, we operated on three distinct datasets: a first set of 24 digitally acquired cases on a GE Senograph 2000D containing 98 images (characteristics: size 1914×2294 pixels, pixel size 0.094 mm, spatial resolution ~5 lp/mm, log pixel-intensity relationship), a second set of 22 digitalized cases on a Lorad Selenia Full Field Digital Mammograph containing 97 images (characteristics: size 3328×4096 pixels, pixel size 0.070 mm, spatial resolution ~7 lp/mm, linear pixel-intensity relationship); and a third set of 103 anonymized individual images containing micro-calcifications clusters digitally acquired on a Fuji CR mammograph (characteristics: size 1770×2370 pixels, pixel size 0.101 mm, spatial resolution ~5 lp/mm, linear pixel-intensity relationship). The combined first two sets contained a number of 10 cases with small (typical dimension ≤ 2 mm) mass lesions showing up in 20 images (10 images for each set) and 28 cases with large sized (typical dimension > 2 mm) mass lesions showing up in 56 images (30 images for the first set and 26 images for the second). The third set contained 73 cases/images with micro-calcifications clusters and 30 reference healthy images. The digital images were all intended for presentation and had a 12-bit greyscale depth. All the digitally acquired images were subsequently stored on a PACS system. The pathologies have been diagnosed and classified by two expeThe mammographic image database used for this study consists of a group of 149 selected cases for a total of 298 images. More specifically, we operated on three distinct datasets: a first set of 24 digitally acquired cases on a GE Senograph 2000D containing 98 images (characteristics: size 1914×2294 pixels, pixel size 0.094 mm, spatial resolution ~5 lp/mm, log pixel-intensity relationship), a second set of 22 digitalized cases on a Lorad Selenia Full Field Digital Mammograph containing 97 images (characteristics: size 3328×4096 pixels, pixel size 0.070 mm, spatial resolution ~7 lp/mm, linear pixel-intensity relationship) ; and a third set of 103 anonymized individual images containing micro-calcifications clusters digitally acquired on a Fuji CR mammograph (characteristics: size 1770×2370 pixels, pixel size 0.101 mm, spatial resolution ~5 lp/mm, linear pixel-intensity relationship). The combined first two sets contained a number of 10 cases with small (typical dimension ≤ 2 mm) mass lesions showing up in 20 images (10 images for each set) and 28 cases with large sized (typical dimension > 2 mm) mass lesions showing up in 56 images (30 images for the first set and 26 images for the second). The third set contained 73 cases/images with micro-calcifications clusters and 30 reference healthy images. The digital images were all intended for presentation and had a 12-bit greyscale depth. rt senior radiologists; all diagnosed pathologies have been further confirmed by histological examination.

The procedure was tested on images belonging to a private anonymous database collected in the Policlinic Hospital of Palermo. Policlinic Hospital is a hospital firm of University of Palermo in which formation, scientific research and health service are well integrated. Policlinic Hospital attests that all research involving humans is carried out in compliance with the Helsinki Declaration and involves appropriate patient consent.

## Results and discussion

**Figure 2:**
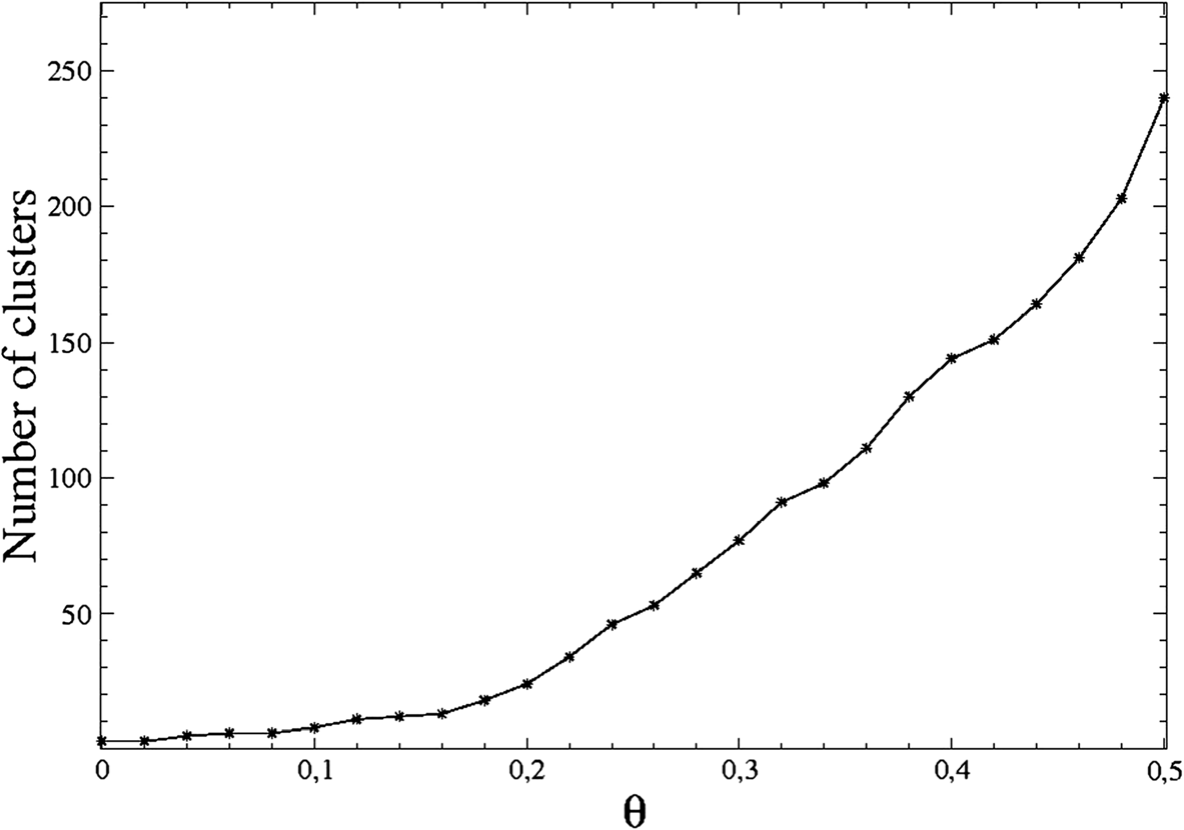
**Short title: typical behavior of the number of clusters as a function of the threshold.** The figure illustrates the increase in the number of clusters for a given image when increasing the threshold parameter θ. The same behavior is displayed by the number of clusters in all the images, with some slight variations of the actual values not affecting the generic shape of the curve. The upper bound has been set θ = 0.5 rather than θ = ln 2 due to the software limitation of the number of clusters.

**Figure 3:**
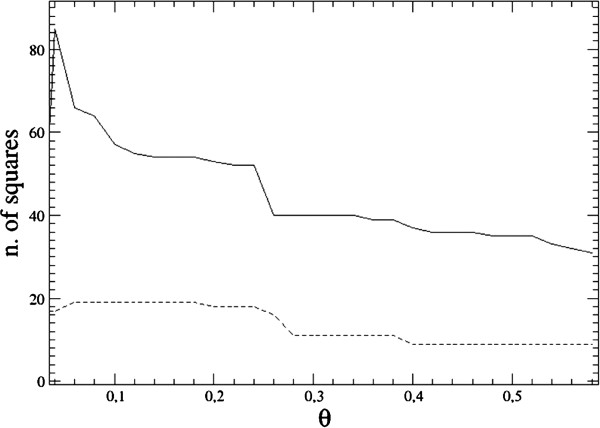
**Size of the two largest clusters as a function of the threshold.** The number of squares contained in the two largest clusters displayed as a function of the threshold parameter θ for a given image. The solid line refers to the first cluster, the dashed line to the second one. The stability ranges are an indicator of meaningfulness for the clusters.

The application of this clustering method yielded a series of interesting results. The most striking consideration is that for a wide range of values of the defining parameters *k* (from the KNN) and *a* (the scale parameter), there appears to be no automatic “best clustering” criterion since the number of clusters exhibits no obvious stationarity when varying θ. The typical dependence of the number of clusters as a function of the threshold θ is depicted in Figure 2. If the clustering is to identify one or a few medically significant regions in the image, it is expected that the corresponding clusters present a minimum of stability also in the number of internal points. The most important clusters in the image actually do exhibit some stability at the varying of the threshold (the internal number of points remains approximately constant on several θ ranges, see e.g. also [26]), but this behavior remains less typical since in a large number of cases, there is no obvious stability subrange or there is no meaningful cluster in the image. In Figure 3 we have represented a typical behavior of the number of points (pixel squares) contained in the two biggest meaningful clusters (other than the three default ones – image background, border pixels and normal internal breast points).

The segmentation algorithm described above displays a fair number of findings in the images containing mass lesions. The “cluster noise” is very large: in fact, at higher threshold levels, most clusters contain actually just one pixel square and show up in internal breast areas characterized by rapid variation of luminosity, typically not far from the breast contour.

**Figure 4:**
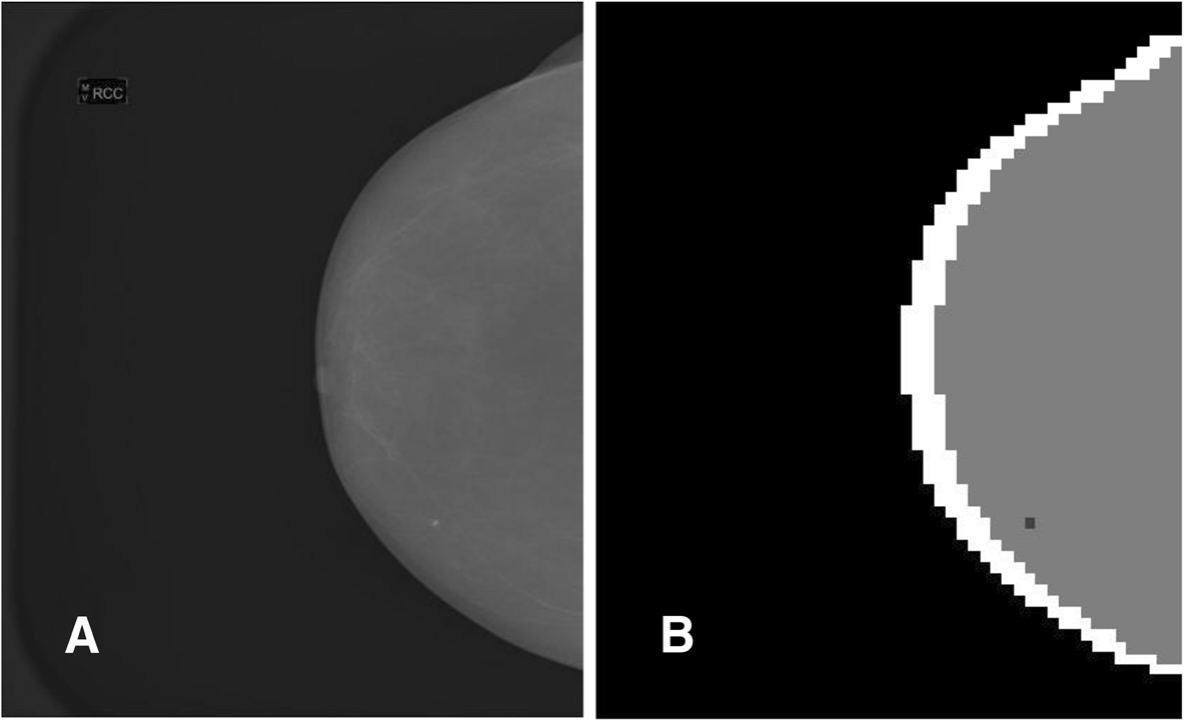
**A small mass lesion showing up in the segmented image for most values of θ.** Panel **A**. The original image. Panel **B**. The segmented image displaying the small mass lesion for a given threshold value θ = 0.1. An essentially similar segmentation shows up for most values of θ.

**Figure 5:**
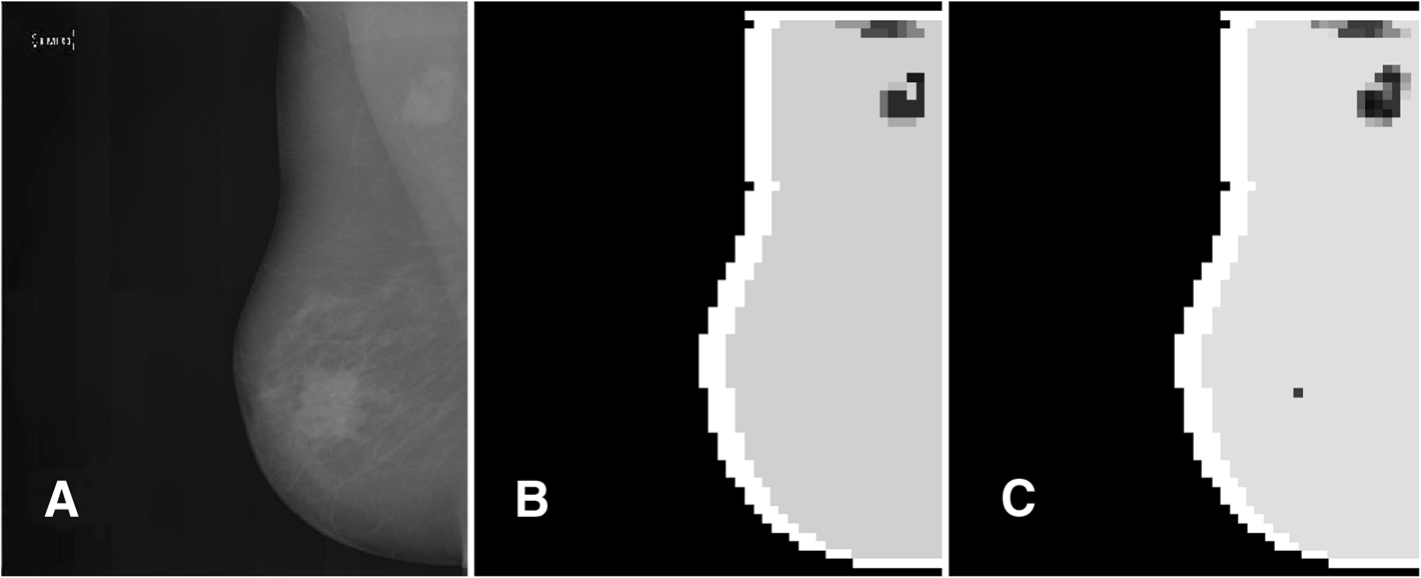
**Large-sized mass objects segmentation.** Large-sized mass objects in a mammography. Panel **A**. The original image. Panel **B**. The image segmented with θ = 0.04. Panel **C**. The image segmented with θ = 0.36. Note the spurious pixel squares near the upper cluster and the lonely pixel square cluster showing up at higher θ.

**Figure 6:**
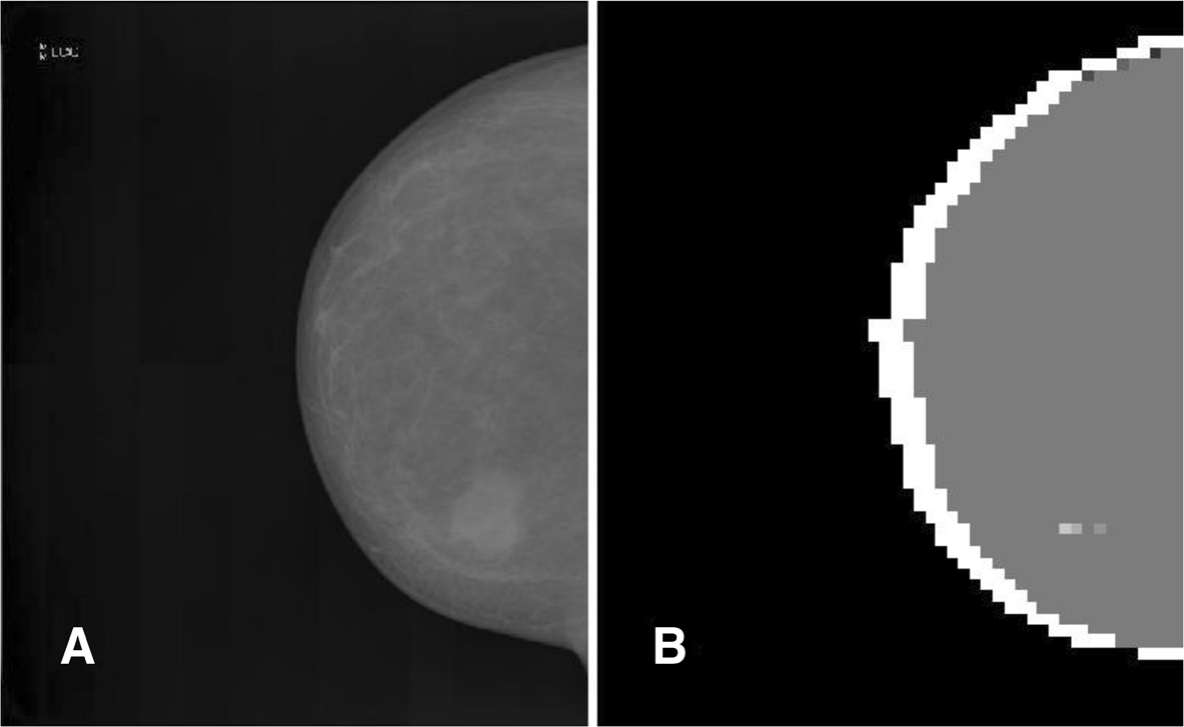
**Large-sized massive opacity segmentation.** Segmentation of a large-sized massive opacity. Panel **A**. The original image. Panel **B**. The image segmented with θ = 0.24. Note the weak correspondence between the segmented internal areas and the actual shape and size of the ROI.

Since there is no clear stability range in the threshold θ, assigning these findings to real ROI for a physician (potential mass lesions) remains a hard task, at least for an automatic system such as a CAD. Basically similar segmented images can be obtained with less effort if clustering only the distance features, which means that the chaotic map clustering algorithm brings little (if any) improvement with respect to the more orthodox and less resource consuming distance-based methods. This result is certainly not surprising since if one excludes their geometrical characteristics, mass lesions usually do not share a mathematically well-defined set of features and the identification of mass ROI is a challenge. In the figures below, some samples illustrating the results of the segmentation algorithm are displayed. Figure 4 exhibits a basic segmentation pattern showing up at most of the threshold values in the case of a small and well-defined mass opacity. Two less satisfactory (according to the physicians opinion) segmentation patterns are shown in the Figures 5 and 6: the first illustrates the occurrence of a potential mass lesion loss within the process, while the second emphasizes the lack of correspondence between the segmented clusters and the shape and size of the actual opacity in the image.

As a general characteristic, the small mass lesions with dimensions of the same order as the size of the pixel square (that is between 1–2 mm), are well identified by the algorithm: practically all of them (15 out of 16, about 94% within this category) show up as isolated point clusters in the segmented image for all but the first threshold values. This behavior may be linked with the important differences in the feature characterization of healthy tissue and small lesions. The meaningfulness of the segmented ROI clusters is ensured by their lack of suspicious clusterized neighboring pixel squares rather than by the variational technique used in the original form of the algorithm’s implementation: the isolation criterion shows no false positive for cluster groups up to four pixel squares immersed in an uniform (healthy tissue) background, not too close to the border of the breast. If one includes also those small clusters connected with the breast border, the correct recognition rate diminishes accordingly and some false positives show up near the border; it’s worthwhile mentioning that in this case the isolation criterion is less operational since all the interesting points cannot be satisfactorily resolved from the spurious pixel squares near the border. No isolated small cluster appears in healthy images.

The results for small mass lesions are summarized in Table 1. In this table, the “Non-Pathologic” label refers to small mass-like objects diagnosed as normal/benign by the physician (4 internal ROI and 4 ROI close to the border). The first row of results contains those ROI segmented as small isolated clusters by the algorithm, while the second row counts the ROI not identified by the algorithm. These results show that the proposed method might be considered as a potential alternative for finding small mass lesions far from the breast border.

**Table 1:** Segmentation of images with pathologic and non-pathologic small mass lesions

**Only internal isolated ROI, actual diagnosis**		**Including breast border isolated ROI, actual diagnosis**	
**Pathologic**	**Non-pathologic**	**Pathologic**	**Non-pathologic**
15 TP	0 (FP)	16 TP	3 (FP)
1 FN	4 (TN)	4 FN	5 (TN)

For large-sized mass lesions extending over an area corresponding to more pixel squares (with typical linear diameters ranging from 3 mm up to about 30 mm), the corresponding segmentation clusters rarely match the shape of the lesion due to the usual non-uniformity of the features over ROI area. About 10% of these lesions (5 out of 56) are matched with an overlap of about 80% by the corresponding segmented clusters; the other large lesions either exhibit overlaps under 30% with their segmented cluster counterparts (33 out of 56), or have no meaningful corresponding cluster associated with them (18 out of 56). On the other hand, in the segmented images, the algorithm introduces often bigger-sized cluster artifacts associated with breast borders or non-pathological denser areas in 32 of the cases, and it is difficult to establish an unambiguous automatic decisional criterion for the degree of meaningfulness of these clusters.

Table 2 summarizes the results for large mass lesions. Overall, these results show that the proposed method is not a good potential alternative for finding large mass lesions.

**Table 2:** Segmentation of large-sized mass lesions (negative images included)

**Large mass lesions**	
**Pathologic**	**Non-pathologic/absent**
38 (= 5 + 33) TP (partial match)	32 (FP)
18 FN	87 (TN)

**Figure 7:**
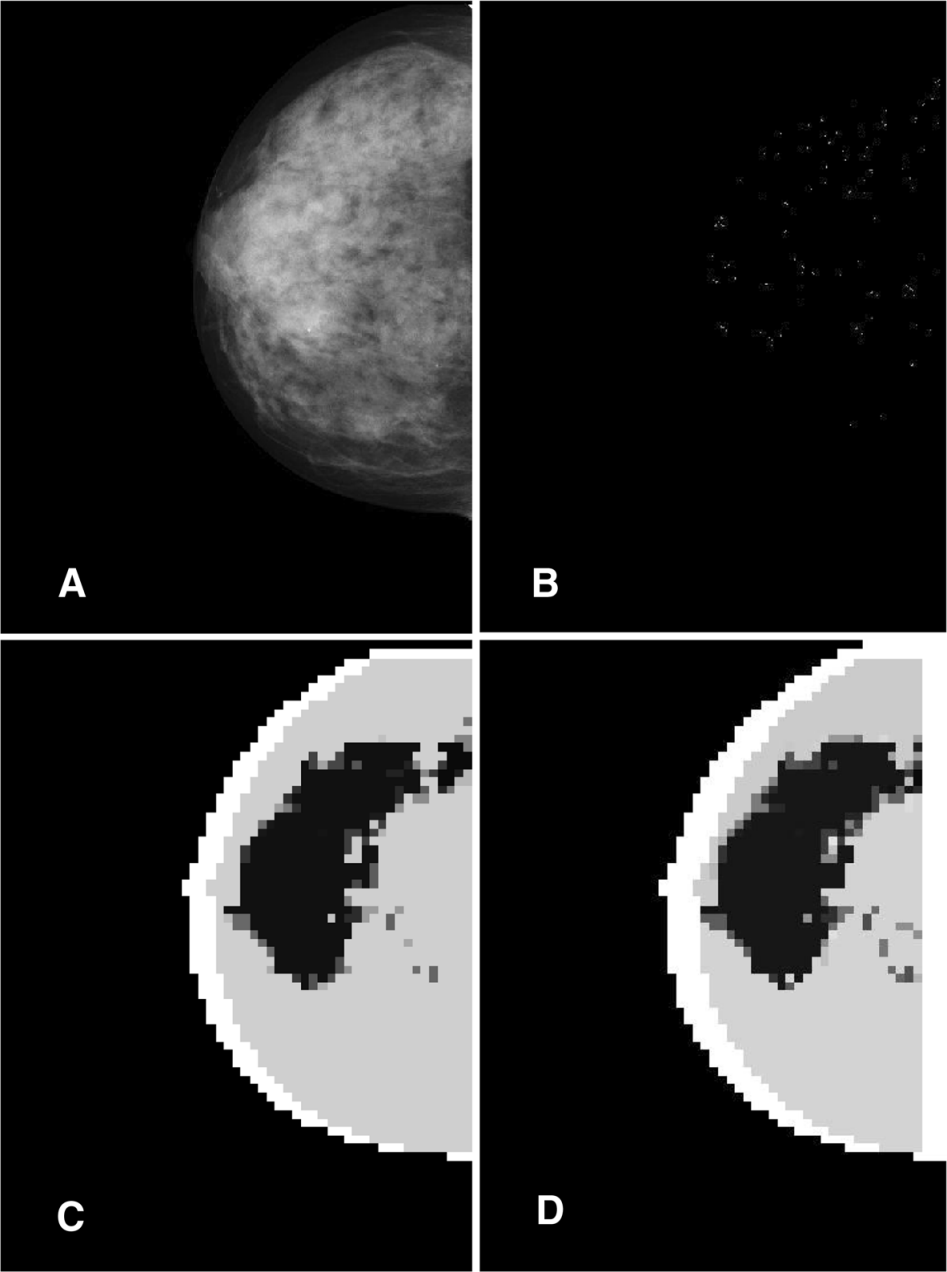
**Segmentation of an image with micro-calcifications.** Segmentation of an image with micro-calcifications. Panel **A**. The original image containing micro-calcifications clusters. Panel **B**. Actual distribution of micro-calcifications as given by the CAD tool. Panel **C**. The segmented image displaying a big cluster for the ROI. Panel **D**. The image segmented with a feature-based scheme.

An interesting behavior is displayed by the images containing micro-calcifications. The parts of the image containing micro-calcifications naturally group in a cluster. The feature analysis thus displays the whole ROI rather than finding individual calcifications, as is visible from Figure 7 above^a^. This result is not surprising due to the well-known reliability of the micro-calcifications characterization through the local features on the image. The overlap of the segmented cluster with the micro-calcifications area varies in the range 10-90% with the peak in the range 30-50%. The agreement is better for denser distributed micro-calcifications.

Due to the distinction naturally arising between small and large/sized mass lesions, one can define an accuracy for each class as *acc*_i_ = (TP_i_ + TN_i_)/( TP_i_ + TN_i_ + FP_i_ + FN_i_ ) where i labels the mass lesion class and the “true/false” are given with respect to the small or large mass lesions. We find thus for small mass lesions an accuracy *acc*_SMALL MASS_ = (195-3-4)/195 ~ 96% (considering also the isolated clusters near the border) and for large mass lesions an accuracy *acc*_LARGE MASS_ = (87 + 38)/195 ~ 64%, in agreement with our previous observations. Of some interest is also the overall accuracy *acc*_MASS_ = 118/195 ~ 60.5% for discriminating between images with generic mass lesions and non-pathological/healthy.

The performance of the method doesn’t exhibit a significant dependence on the database: the accuracy results restricted to the first set are *acc*_SMALL MASS_ = 95/98 ~ 97% and *acc*_LARGE MASS_ = 61/98 ~ 62%, while on the second set one has *acc*_SMALL MASS_ = 93/97 ~ 96% and *acc*_LARGE MASS_ = 64/97 ~ 66%.

Concerning the 103 images in the micro-calcifications dataset, 3 of the healthy images present an internal contiguous cluster similar to the one underlying a part of the positives. The remaining 73 positive ones do exhibit internal “big” clusters distributed according to the following overlaps:

If the overlap in the segmented image with micro-calcifications is enough consistent (our tests show that an overlap of at least 30% with the denser micro-calcification area constitutes already a safe indication) to trigger a further analysis in an automatic system, the internal segmentation cluster will contain most of the micro-calcifications and may be used as a relevant investigation starting point. It should be mentioned at this point that the feature-only based approach produces an essentially similar segmentation pattern. Therefore, the chaotic map clustering of the mammographic images containing micro-calcifications brings no extra information with respect to this alternative method.

Assuming that overlaps up to 25% are not pathology-conclusive, the number of false negatives is essentially given by the sum of the first three terms in Table 3. On the other hand, the false positives are the 3 healthy images segmented with the internal big cluster, therefore one may estimate an accuracy *acc*_MICRO_ = (103-23-3)/103 ~ 75%.

**Table 3:** Overlap of the cluster with the ROI for micro-calcifications images

Overlap range	0-5%	5-15%	15-25%	25-35%	35-45%	45-55%	55-65%	65-75%	75-85%	85-95%
# of images	11	4	8	12	12	9	2	4	6	5

## Conclusion

The non-parametric chaotic map clustering of the mammographic images has been considered here as stand-alone segmentation approach, mainly from an applicability point of view. The ultimate goal of applying such a segmentation method to the medical mammographic images is the potential performance improvement of an automatic detection system based on it. As discussed, the specific aspect of mammographic segmentation which remains a non-trivial challenge is the segmentation of mass lesions, while the identification of micro-calcifications with this new algorithm hardly could lead to any spectacular breakthrough advance (micro-calcifications detection rates of about 94% with 6.25% of false positives and 2% false negatives were already reported more than a decade ago, see [40]).

At this stage of the analysis, the results obtained do allow some general conclusions concerning the valuable applicability of the chaotic map algorithm for the segmentation of mammographic images, in an efficient automatic work-flow, in comparison with the results obtained by alternative methods as those used by present day commercial CAD systems. While many (about 90%) of the mass lesions are either lost or appear with wrong sizes, shapes and as neighboring independent clusters (see Figures 5 & 6 above), most of the smaller ones show up conveniently as internal clusters in the segmented images. Indeed, about 94% of the small lesions more than 6 mm away from the border were correctly segmented by the algorithm; the true positive rate decreases to 80% if the smaller mass lesions near the breast border are included. This fact looks especially important when considering that the small lesions are usually less easily identifiepathologic cases cod than the extended ones, and the support of an automatic CAD system is more useful in their case. On the other hand, one has to keep in mind that the important number of “parasite” clusters with no medical significance adds a further complication in correctly evaluating the output of the segmentation algorithm which the stability analysis cannot eliminate.

Concerning the micro-calcifications, the chaotic maps segmentation process gives interesting and peculiar results. In about 2/3 of the pathologic cases considered here, the algorithm provides an useful shape of the region with denser micro-calcifications. While these results are still not significantly edging the ones derived from simple feature analysis, the algorithm may be used as alternative check in a more complex workflow.

Due to the particularities of the mammographic images, we conclude that the chaotic map clustering algorithm should not be used as unique stand-alone method of segmentation. It is rather the joint use of this method along with other segmentation techniques that could be successfully used for increasing segmentation performance and providing extra information for subsequent analysis stages such as the classification of the segmented ROI.

## Endnote

^a^The CAD tool used for computing the position of micro-calcifications is CyclopusCAD Mammo® produced by CyclopusCAD srl.

## Competing interests

• We have not received in the past five years reimbursements, fees, funding, or salary from an organization that may in any way gain or lose financially from the publication of this manuscript, either now or in the future.

• We do not hold any stocks or shares in an organization that may in a ny way gain or lose financially from the publication of this manuscript, either now or in the future.

• We do not hold or we are not currently applying for any patents relating to the content of the manuscript. We have not received reimbursements, fees, funding, or salary from an organization that holds or has applied for patents relating to the content of the manuscript.

• We have not any other financial competing interests. Biltawi, M.; Al-Najdawi, N.; Tedmori, S.; Mammogram enhancement and segmentation methods: classification, analysis, and evaluation. The 13th international Arab Conference on Information Technology, December 2012.

• There are not any non-financial competing interests (political, personal, religious, ideological, academic, intellectual, commercial or any other) to declare in relation to this manuscript.

## Authors’ contributions

MI and DC conceived of the study, carried out the Clustering algorithm implementation and performed the statistical analysis and drafted the manuscript. FF and GR conceived of the study, participated in the design and coordination of the manuscript, and helped to its draft. All authors read and approved the final manuscript.

## Pre-publication history

The pre-publication history for this paper can be accessed here:

http://www.biomedcentral.com/1471-2342/14/12/prepub
